# Long non‐coding RNA CASC15 regulates gastric cancer cell proliferation, migration and epithelial mesenchymal transition by targeting CDKN1A and ZEB1

**DOI:** 10.1002/1878-0261.12187

**Published:** 2018-05-09

**Authors:** Qiong Wu, Shihao Xiang, Jiali Ma, Pingping Hui, Ting Wang, Wenying Meng, Min Shi, Yugang Wang

**Affiliations:** ^1^ Department of Gastroenterology Tongren Hospital Shanghai Jiao Tong University School of Medicine China

**Keywords:** cancer susceptibility candidate 15, CDKN1A, epithelial‐to‐mesenchymal transition, gastric cancer, ZEB1

## Abstract

Long non‐coding RNA (lncRNA) is responsible for a diverse range of cellular functions, such as transcriptional and translational regulation and variance in gene expression. The lncRNA CASC15 (cancer susceptibility candidate 15) is a long intergenic non‐coding RNA (lincRNA) locus in chromosome 6p22.3. Previous research shows that lncRNA CASC15 is implicated in the biological behaviors of several cancers such as neuroblastoma and melanoma. Here, we aimed to explore in detail how CASC15 contributes to the growth of gastric cancer (GC). As predicted, the expression of CASC15 was enriched in GC tissues and cell lines as compared with healthy tissues and cells using qRT‐PCR. The Kaplan–Meier method was used to demonstrate that high expression of CASC15 is linked to a poor prognosis for patients suffering from GC. Additionally, functional experiments proved that the down‐ or up‐regulation of CASC15 inhibited or facilitated cell proliferation via the induction of cell cycle arrest and apoptosis, and also suppressed or accelerated cell migration and invasion by affecting the progression of the epithelial‐to‐mesenchymal transition (EMT). *In vivo* experiments showed that the knockdown of CASC15 lessened the tumor volume and weight and influenced the EMT process. This was confirmed by western blot assays and immunohistochemistry, indicating impaired metastatic ability in nude mice. CASC15 involvement in the tumorigenesis of GC occurs when CASC15 interacts with EZH2 and WDR5 to modulate CDKN1A in nucleus. Additionally, the knockdown of CASC15 triggered the silencing of ZEB1 in cytoplasm, which was shown to be associated with the competitive binding of CASC15 to miR‐33a‐5p.

**Abbreviations**
GC: gastric cancer
GES‐1: gastric epithelial cell line
IHC: immunohistochemistry
IP: immunoprecipitation
lincRNA: long intergenic non‐coding RNA
lncRNA: long non‐coding RNA
MTT: 3‐(4,5‐dimethylthiazole‐2‐yl)‐2,5‐diphenyltetrazolium bromide
NF‐κB: nuclear factor kappa B
RIP: RNA immunoprecipitation
siRNA: short interfering RNA
SPF: specific pathogen‐free
TCGA: The Cancer Genome Atlas
TNM: tumor, node and metastasis

## 1 Introduction

As the most common malignant human tumor, gastric cancer (GC) is the second primary cause of cancer‐associated mortalities around the world, in spite of great advances in diagnosing and treating human malignancies (Ferlay *et al*., 2015; The Cancer Genome Atlas Research Network, 2014). Metastasis is mainly responsible for the high number of fatalities, greatly preventing efficient treatment, although there have been great advances in the medical technology for GC (Gupta and Massague, 2006). Thus, it is critically important to identify key genes and to understand their molecular mechanisms for GC prognosis and therapy. Since the early 2000s, a large amount of research has been focused on genes capable of coding proteins. A cluster of genes – AP‐1, nuclear factor kappa B (NF‐κB), SNAI1, Sp1, EGR, CREB and ATF – have been noted to engage in GC growth (Abdel‐Latif *et al*., 2009; Mitsuno *et al*., 2001; Pradeep *et al*., 2004; Redlak *et al*., 2008; Safe and Abdelrahim, 2005; Schewe *et al*., 2003; Szalad *et al*., 2009). However, up to now, the potential molecular mechanism of the malignancy of GC remains obscure.

With the great progress made in gene sequencing and analysis techniques, it has been discovered that 98% of gene sequencing is incapable of coding protein, and 87% of genome sequences develop into biochemical transcripts (Niu and Jiang, 2013). Lacking protein‐coding ability, long non‐coding RNA (lncRNA) possesses > 200 nt, serving as decoys and facilitating both proximal and distal macromolecular interactions (Liu *et al*., 2012; Okazaki *et al*., 2002; Ulitsky and Bartel, 2013; Zhang *et al*., 2016b). There is also increasing evidence that lncRNA participate in a series of cellular processes by influencing diverse expression of protein, DNA and RNA, and their mutual cooperation (Mercer *et al*., 2009; Ponting *et al*., 2009; Wang and Chang, 2011; Wang *et al*., 2014; Wilusz *et al*., 2009). The relation between abnormally expressed lncRNA and the invasive/migratory abilities of GC cells has recently been discussed (Sun *et al*., 2016b; Wiestler *et al*., 2014; Zhang *et al*., 2016a; Zhao *et al*., 2017). It is thus critical to determine the biological functions of more lncRNA for the diagnosis and treatment of GC.

Chromosome 6p22 [cancer susceptibility candidate 15 (CASC15)] was recently defined as a neuroblastoma susceptibility locus (Russell *et al*., 2015) and the chromosome 6p22.3 CASC15 long intergenic non‐coding RNA (lincRNA locus) was discovered to be able to gain a genomic segment in melanoma (Lessard *et al*., 2015). However, its biological contributions to the tumorigenesis of GC are as ye unclear. In this paper, high expression of CASC15 was associated with poor prognosis of GC patients. In addition, functional assays were applied to test the effects of silenced vs. strengthened CASC15 on the biological behaviors in GC, demonstrating that the over‐ or underexpression of CASC15 remarkably suppressed or boosted the cell migration and invasion. In addition, western blot and immunofluorescence showed that the protein levels of epithelial marker (E‐cadherin) and mesenchymal marker (N‐cadherin) were regulated by the up‐ or down‐regulated CASC15, demonstrating that CASC15 could affect migratory and invasive abilities of GC cells by influencing epithelial‐to‐mesenchymal transition (EMT) progression. The *in vivo* experiments showed that the knockdown of CASC15 could impair the tumor volume and weight in nude mice, as well as influencing EMT process, as confirmed by western blot and immunohistochemistry (IHC) assays. Subsequently, mechanistic assays proved that CASC15 engaged in the tumorigenesis of GC through interaction with EZH2 and WDR5 to modulate CDKN1A in the nucleus. At the same time, it was discovered that the knockdown of CASC15 triggered the silencing of ZEB1 in cytoplasm, which was attributed to the competitive sponge of CASC15 with miR‐33a‐5p.

Here, we hypothesized that CASC15‐miR‐33a‐5p‐CDKN1A/ZEB1 axis would be a novel pathway in gastric cancer.

## 2 Materials and methods

### 2.1 Tissue specimens

A total of 88 GC patients who had undergone surgery in the Tongren Hospital, Shanghai Jiao Tong University School of Medicine, were included in this study. Neither chemotherapy nor radiotherapy before surgery had been conducted in any of the patients. The University Ethics Committee granted approval for this study. All patients gave their personal informed consent. Clinical characteristics are presented in Table 1.

**Table 1:** Correlation between CASC15 expression and clinical features (*n *=* *88). Low/high by the sample mean. Pearson chi‐square test

Variable	CASC15 expression		*P*‐value
	Low	High	
Age			
> 60	8	12	0.449
≤ 60	35	33	
Gender			
Male	28	31	0.821
Female	15	14	
TNM stage			
I + II	22	13	0.049*
III + IV	21	32	
Tumor invasion			
T1 + T2	30	14	0.001**
T3 + T4	13	31	
Lymph node metastasis			
N0 + N1	30	13	< 0.001***
N2 + N3	13	32	
Distant metastasis			
M0	26	16	0.032*
M1	17	29	
Tumor differentiation			
Well + Moderate	41	40	0.435
Poor	2	5	

*P <* *0.05, **P <* *0.01 and ***P <* *0.001 were considered statistically significant.

### 2.2 Cell lines and cell culture

Six GC cell lines (BGC‐823, AGS, SGC‐7901, NCI‐N87, MKN‐45 and MKN‐28) and the healthy human gastric epithelial cell line (GES‐1) were provided by the Institute of Biochemistry and Cell Biology of the Chinese Academy of Sciences. All cell lines were kept in RPMI‐1640 medium with the addition of 10% FBS (Wisent, Ottawa, ON, Canada), 100 U·mL^−1^ penicillin, and 100 mg·mL^−1^ streptomycin (Invitrogen, Carlsbad, CA, USA) in humid conditions with 5% CO_2_ at 37 °C.

### 2.3 RNA extraction and qRT‐PCR

TRIzol reagent (Invitrogen) was used to extract RNA from tissues or cultured cells. For qRT‐PCR, RNA was inversely transcribed into cDNA with a Reverse Transcription Kit (Takara, Tokyo, Japan). qRT‐PCR was analyzed using SYBR Green (Takara). The results were normalized to GAPDH. The primers used for qRT‐PCR were: CASC15 (F) 5′‐CACACGCATGGAAAACCCAG‐3′ and (R) 5′‐GAGGACCTGAGCTGTAAGCC‐3′; CDKN1A (F) 5′‐ AAGTCAGTTCCTTGTGGAGCC‐3′ and (R) 5′‐ GGTTCTGACGGACATCCCCA‐3′; EZH2 (F) 5′‐GGCTCCTCTAACCATGTTTACAACT‐3′ and (R) 5′‐ AGCGGTTTTGACACTCTGAACTAC‐3′; WDR5 (F) 5′‐ GCCTACACCTGTGAAGCCAAAC‐3′ and (R) 5′‐ GAATCTGACGACCAGGCTACAT‐3′; miR‐33a‐5p (F) 5′‐GATCCTCAGTGCATTGTAGTTGC‐3′ and (R) 5′‐CTCTGTCTCTCGTCTTGTTGGTAT‐3′; ZEB1 (F) 5′‐AAGTGGCGGTAGATGGTAATGT‐3′ and (R) 5′‐AAGGAA GACTGATGGCTGAAAT‐3′; GAPDH (F) 5′‐AGAAGGCTGGGGCTCATTTG‐3′ and (R) 5′‐ AGGGGCCATCCACAGTCTTC‐3′. The 2^−ΔΔCt^ method was applied to carry out three independent experiments.

### 2.4 Cell transfection

GENEWIZ was used to synthesize the cDNA of lncRNA CASC15, which was then was cloned into pcDNA3.1. DNA fragments of CASC15‐shRNA were also constructed by GENEWIZ and fixed into the *Bgl*II/*Hind*III sites following annealing. The cDNA of ZEB1 was extracted by RT‐PCR, followed by the cloning into the *Hind*III/*EcoR*I sites of pcDNA3.1. Lipofectamine 2000 was applied to conduct transfections in accordance with the manufacturer's guidelines (Invitrogen). We then selected cells with stable cloning.

### 2.5 Transwell assays

In the migratory ability assay, we placed cells (4 × 10^5^) into the upper compartment (Millipore, Billerica, MA, USA) using the non‐coated membrane. For the invasive ability assay, we prepared Matrigel (BD Biosciences, New York, NY, USA) in transwell inserts for 30 min at 37 °C. In transwell migration and invasion experiments, cells were put into the top compartment of the medium lacking serum; the lower compartment was filled with 10% FBS and served as a chemoattractant. After 36 h of cultivation, non‐migratory/invasive cells via the pores were lightly separated using cotton swabs. All of the cells stained with crystal violet and then were divided into five fields with an inverted microscope. Three independent assays were carried out.

### 2.6 *In vivo* assays and IHC

Male nude mice weighing 20 g, born 4 weeks earlier, were conserved in an environment with air‐conditioning and specific pathogen‐free (SPF). Ten such mice were divided into two groups at random, which were injected with sh‐CASC15‐ or empty vector‐transfected AGS cell (1 × 10^6^ cells per mouse) via tail intravenous injection. After 45 days, the above mice were killed. All animal studies were approved by the Animal Welfare and Research Ethics Committee at Tongren Hospital, Shanghai Jiao Tong University School of Medicine, and all protocols were performed specifically on the basis of the Guide for the Care and Use of Laboratory Animals. The primary tumors were excised, paraffin‐embedded and formalin‐fixed, followed by hematoxylin and eosin (HandE) staining and immunostaining to analyze the expression of Ki‐67, in accordance with the manufacturer's instructions.

### 2.7 Western blot assay

The protein lysates for all cells were split using 10% SDS/PAGE. Later, they were shifted onto 0.22‐μm nitrocellulose (NC) membranes (Sigma, San Francisco, CA, USA) and then incubated with specific antibodies. Anti‐EZH2 (cat#: ab186006) and WDR5 (cat#: ab56919; Bioworld Technology, Saint Paul, MN, USA), anti‐E‐cadherin (cat#: ab1416), anti‐N‐cadherin (cat#: ab18203; Cell Signaling Technology, Boston, MA, USA), vimentin (cat#: ab8978; Santa Cruz Biotechnology, Santa Cruz, CA, USA) and anti‐GAPDH (Sigma‐Aldrich) were used as controls. Protein was detected with Super Signal Chemiluminescence Substrate (Pierce, Thermo Scientific, Waltham, MA, USA).

### 2.8 Subcellular fractionation location

Nuclear and cytoplasmic fractions were segregated using PARIS Kit (Life Technologies, Carlsbad, CA, USA) based on the manufacturer's instructions. RNA was prepared for the following reverse transcription reaction and real‐time PCRs (SYBR Premix Ex Taq; TaKaRa).

### 2.9 ChIP assays

ChIP assays were conducted using the ChIP Kit in accordance with the manufacturer's instructions (Millipore). Anti‐H3K27me3/H3K4 trimethylation (Millipore) was applied for immunoprecipitations (IP), and normal mouse IgG was used as the negative control. The primer sequences from CDKN1A promoter were prepared for PCR experiments, covering the upstream transcriptional sites of CDKN1A gene.

### 2.10 RIP assays

RNA immunoprecipitation (RIP) assays were designed by means of a Magna RIP RNA‐Binding Protein IP Kit (Millipore) on the basis of the manufacturer's guidance. Antibodies for EZH2 (cat#: ab186006) and WDR5 (cat#: ab56919) were acquired from Abcam. RT‐PCR was used to detect the co‐precipitated RNA.

### 2.11 Dual luciferase reporter assay

PCR was performed to amplify ZEB1 cDNA segments including miR‐33a‐5p binding sites, followed by the cloning into pGL3 vector (Promega, Madison, WI, USA). We kept cells in the 24‐well plate for 24 h of cultivation, followed by transfection with miR‐33a‐5p mimick or respective controls. The luciferase reporters were treated with Lipofectamine 2000 in accordance with the manufacturers' instructions. After 48 h, cells were harvested, followed by firefly and renilla luciferase activity detection using a Glomax 2020 Single Tube Luminometer (Promega). The light intensity from firefly luciferase was normalized to the renilla luciferase signal. All assays were carried out independently in triplicate.

### 2.12 Statistical analysis

The experimental results are presented as mean ± SD. The statistical differences among different groups were analyzed by Student's ‘*t*’‐test. Spearman's correlation analysis was used to detect the relationship among genes. A *P*‐value < 0.05 was considered statistically significant. graphpad prism 5 (GraphPad Software, La Jolla, CA, USA) or spss 20.0 (SPSS, Chicago, IL, USA) was used for all statistical analyses.

## 3 Results

### 3.1 CASC15 indicates poor prognosis for GC patients

**Figure 1:**
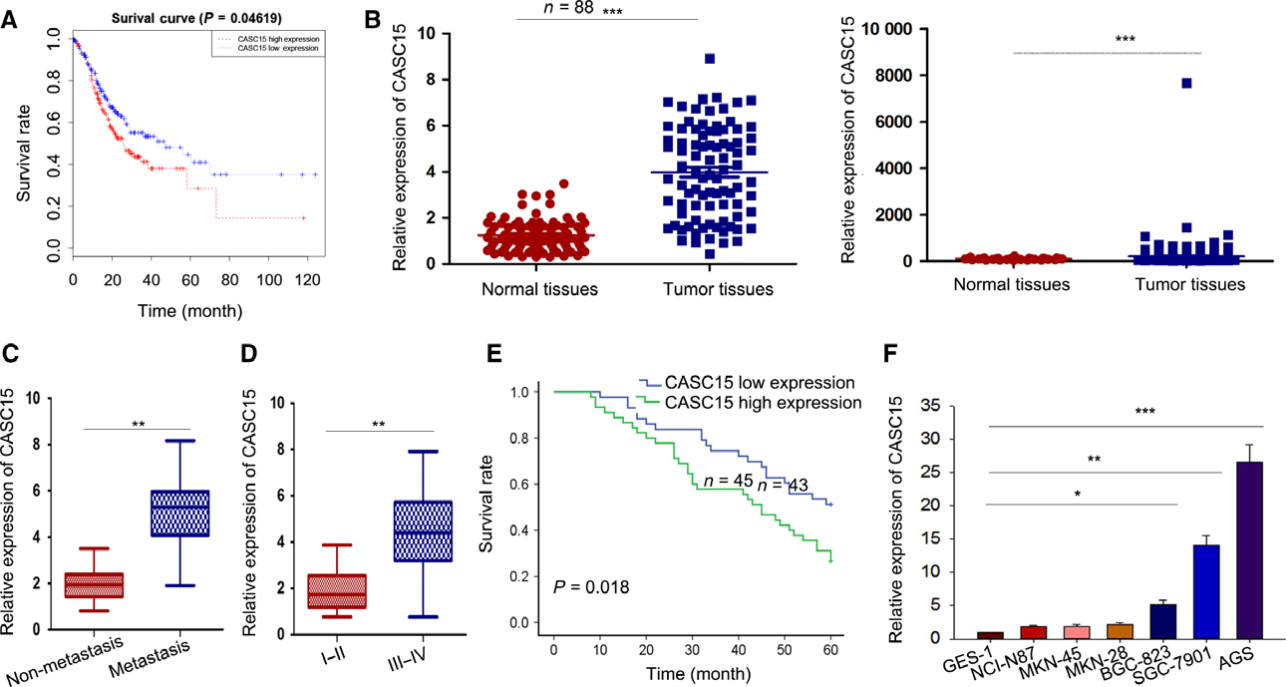
Highly expressed CASC15 is associated with poor survival in GC patients. (A) On the basis of TCGA data, a high level of CASC15 was positively correlated with poorer overall survival outcome. (B–D). qRT‐PCR and TCGA analysis demonstrated that CASC15 was obviously up‐regulated in GC tissues compared with normal tissues (B). Tissues with distant metastasis presented a higher expression level of CASC15 than those without distant metastasis (C). CASC15 was significantly correlated with the TNM stage of GC (D). (E) Kaplan–Meier analysis (log‐rank test) showed that higher CASC15 was associated with poorer overall survival. (F) qRT‐PCR was used to demonstrate that the expression of CASC15 was significantly increased in the GC cell lines. Error bars represent the mean ± SD of at least three independent experiments. *P <* *0.05, **P <* *0.01 and ***P <* *0.001 vs. control group.

By analyzing The Cancer Genome Atlas (TCGA) data, we revealed that highly expressed CASC15 was positively related to poorer overall survival outcome (Fig. 1A), implying that CASC15 has a powerful prognostic value for GC patients and a potential oncogenic role in GC. We then explored the expression of CASC15 in 88 paired GC tissues and matched healthy tissues by qRT‐PCR and TCGA analysis, both normalized to GAPDH. Based on qRT‐PCR and TCGA analysis, CASC15 was obviously up‐regulated in GC tissues (*P *<* *0.001; Fig. 1B). Tissues with distant metastasis presented higher expression levels of CASC15 than those without distant metastasis (*P *<* *0.001; Fig. 1C). In addition, highly expressed CASC15 was markedly related to the tumor, node and metastasis (TNM) stage of GC (*P < 0.05, **P < 0.01; Fig. 1D).

To explore the clinicopathological role of CASC15 in GC, we classified 88 patients into two groups on the basis of the mean value. The association between CASC15 expression and clinicopathological features is shown in Table 1. Patients with up‐regulated CASC15 suffered from tumor invasion (*P *= 0.001), lymph node metastasis (*P *< 0.001), distant metastasis (*P *= 0.032) and higher TNM stage (*P *= 0.049). However, the association of CASC15 expression with age, gender and tumor differentiation was unclear. Furthermore, the Kaplan–Meier method (log‐rank test) indicated that higher expression of CASC15 predicted poorer overall survival (*P *= 0.018; Fig. 1E).

In addition, to assess whether CASC15 could be used to predict GC development, we applied univariate and multivariate analyses. Univariate analysis indicated that distant metastasis (*P *= 0.048), TNM stage (*P *= 0.006) and CASC15 expression (*P *= 0.003) might influence the survival rate of GC patients (Table 2). In contrast, multivariate analysis, applying a Cox proportional hazards model, demonstrated that only distant metastasis (*P *= 0.048) and CASC15 expression (*P *= 0.003) are impact factors in GC patients (Table 2). Finally, by employing qRT‐PCR analysis, we profiled the level of CASC15 in a panel of GC cells and one human gastric epithelial cell; the results showed that CASC15 was much more enriched in GC cells than in the healthy cell (Fig. 1F). These investigations indicated that enhanced CASC15 might be involved in the progression of GC.

**Table 2:** Multivariate analysis of prognostic parameters in patients with CASC15 by Cox regression analysis. Proportional hazards method analysis showed a positive, independent prognostic importance of CASC15 expression (*P *= 0.003)

Variable	Category	*P*‐value
Age	>60	0.207
	≤60	
Gender	Male	0.659
	Female	
TNM stage	I + II	0.006**
	III + IV	
Tumor invasion	T1 + T2	0.699
	T3 + T4	
Lymph node metastasis	N0 + N1	0.229
	N2 + N3	
Distant metastasis	M0	0.048*
	M1	
Tumor differentiation	Well + moderate	0.862
	Poor low	
CASC15	High	0.003**

*P < 0.05; **P < 0.01 were considered statistically significant.

### 3.2 CASC15 regulates GC cell proliferation, migration and EMT

**Figure 2:**
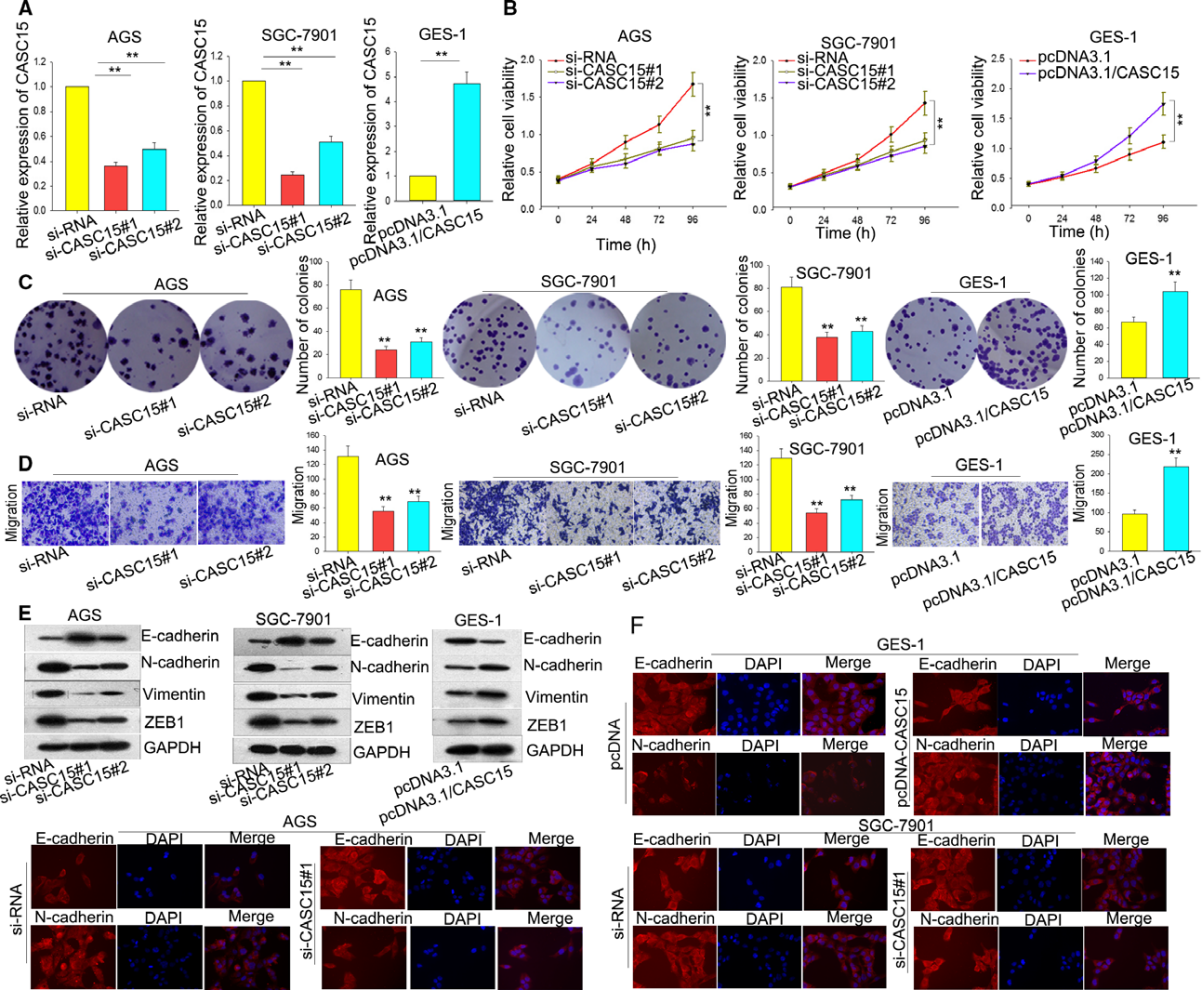
CASC15 regulates GC cell proliferation, migration and EMT. (A) AGS and SGC7901 cells transfected with si‐CASC15 exhibited a relatively high expression of CASC15 (left). GES‐1 cell transfected with pcDNA‐CASC15 showed a relatively low level of CASC15 (right). (B,C) MTT and colony formation assays revealed that the silencing of CASC15 inhibited cell viability and clonogenic survival, whereas overexpression of CASC15 enhanced cell viability and clonogenic survival, compared with the control group. (D) Transwell assays demonstrated that the knockdown of CASC15 caused clearly inhibited cell migration, whereas overexpression of CASC15 caused the opposite. (E) Western blot assays revealed that the silencing of CASC15 decreased the levels of the mesenchymal markers but increased the level of the epithelial marker; overexpression of CASC15 caused the opposite effect. (F) Immunofluorescence staining revealed that the dysregulated CASC15 changed the distribution of E‐cadherin and N‐cadherin in the AGS cell. Error bars represent the mean ± SD of at least three independent experiments. **P <* *0.01 vs. control group.

To clarify the functions of CASC15, we first designed two independent short interfering RNA (siRNA) targeting CASC15; these were transfected into AGS and SGC7901 cells, exhibiting a relatively high level of CASC15 (Fig. 2A, left). To avoid the off‐target effects of siRNA, we constructed the overexpressed vector of CASC15, which was transfected into GES‐1 cell, demonstrating a relatively low level of CASC15 (Fig. 2A, right). Observations from 3‐(4,5‐dimethylthiazole‐2‐yl)‐2,5‐diphenyltetrazolium bromide (MTT) assays revealed that silenced CASC15 significantly inhibited cell viability compared with the control cells, whereas the opposite effect was seen in the GES‐1 cells transfected with overexpressed CASC15 (Fig. 2B).

Cell cloning experiments demonstrated that silenced CASC15 impaired cell colonial ability, whereas overexpressed CASC15 boosted the number of clones (Fig. 2C). To verify the effect of silenced CASC15 on cell proliferation, we performed flow cytometric analyses to examine the effect of the knockdown or overexpression of CASC15 on cell cycle and apoptosis. As presented in Supporting Information Fig. S1A, the knockdown of CASC15 induced marked cell cycle arrest, causing far more cells to arrest in the G0/G1 phase compared with the negative control. Conversely, overexpression of CASC15 accelerated cell cycle progression, triggering many more cells at the S phase. Similarly, compared with the negative control, silenced CASC15 increased the number of apoptotic cells, whereas up‐regulation of CASC15 decreased the number of apoptotic cells (Fig. S1B).

Consistent with the flow cytometry analysis of apoptosis, western blot showed that the levels of proteins associated with apoptosis (caspase 3 and caspase 9) were enhanced by knockdown of CASC15 but impaired by up‐regulation of CASC15, both compared with the negative control (Fig. S1C). Subsequently, the migration assay revealed that in comparison with the negative control, cell migratory ability was clearly inhibited by the silencing of CASC15 but, conversely, was increased in cells transfected with overexpressed CASC15 (Fig. 2D). Furthermore, western blot assays demonstrated that silencing of CASC15 decreased the levels of the mesenchymal markers N‐cadherin, vimentin and ZEB1, but increased the level of the epithelial marker E‐cadherin. In contrast, overexpression of CASC15 was able to increase the levels of N‐cadherin, vimentin and ZEB1, but decrease the level of E‐cadherin (Fig. 2E). Consistent with this result, immunofluorescence staining revealed that the dysregulated CASC15 changed the distribution of E‐cadherin and N‐cadherin in the AGS cell (Fig. 2F). These investigations indicated that CASC15 was involved in the progression of GC.

### 3.3 CASC15 regulates GC tumorigenesis *in vivo*

**Figure 3:**
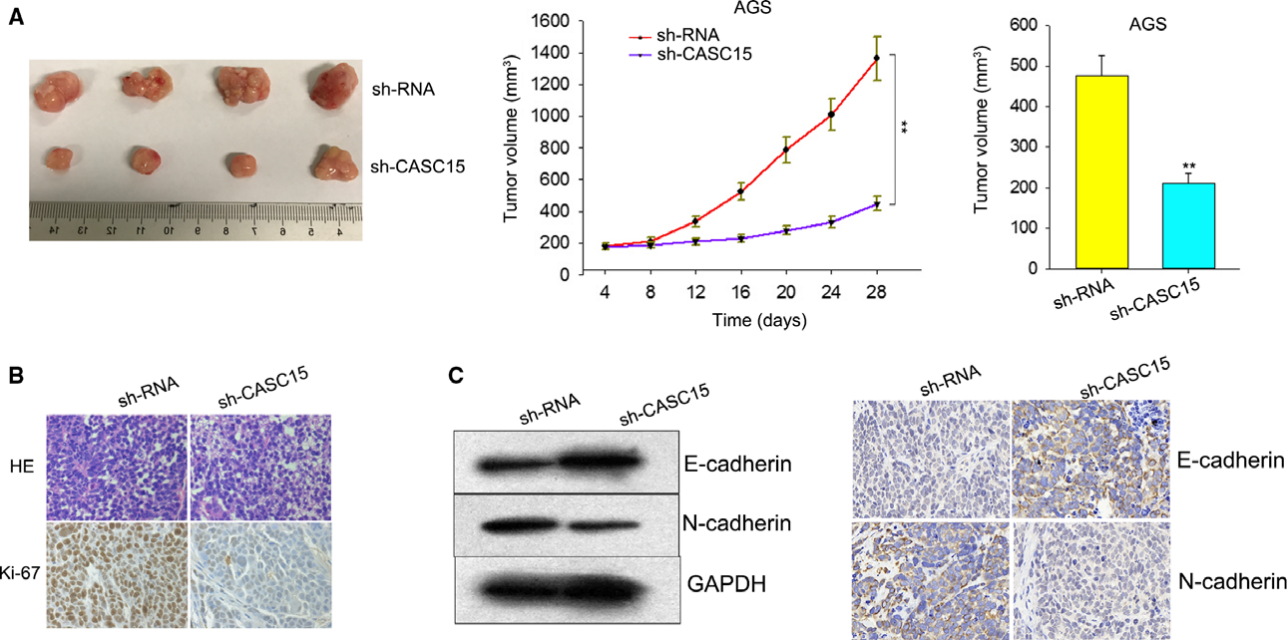
CASC15 regulates GC tumorigenesis *in vivo*. (A) The sh‐CASC15 transfection decreased the tumor volume and weight *in vivo*. (B) The sh‐CASC15‐transfected AGS cell exhibited a weakened positivity for Ki‐67 compared with control cells. (C) Western blot revealed that silenced CASC15 was able to reduce significantly the level of N‐cadherin but increase the level of E‐cadherin. Error bars represent the mean ± SD of at least three independent experiments. **P <* *0.01 vs. control group.

To further confirm whether CASC15 could affect the tumorigenesis of GC *in vivo*, a sh‐CASC15‐ or control vector‐transfected AGS cell was intravenously injected into the tails of selected mice. The tumors derived from the sh‐CASC15‐transfected cell were markedly smaller than that from the control vector‐transfected cell, showing a marked reduction of tumor volume and weight (Fig. 3A). Positive Ki‐67 in the sh‐CASC15‐interfered AGS cell was weaker than that in the negative control cell (Fig. 3B). Moreover, western blot and IHC revealed that silenced CASC15 markedly decreased the expression of N‐cadherin but increased the expression of E‐cadherin (Fig. 3C). The *in vivo* data complemented the description of the oncogenetic role of CASC15.

### 3.4 CASC15 modulates CDKN1A through interaction with EZH2 and WDR5 in the nucleus

**Figure 4:**
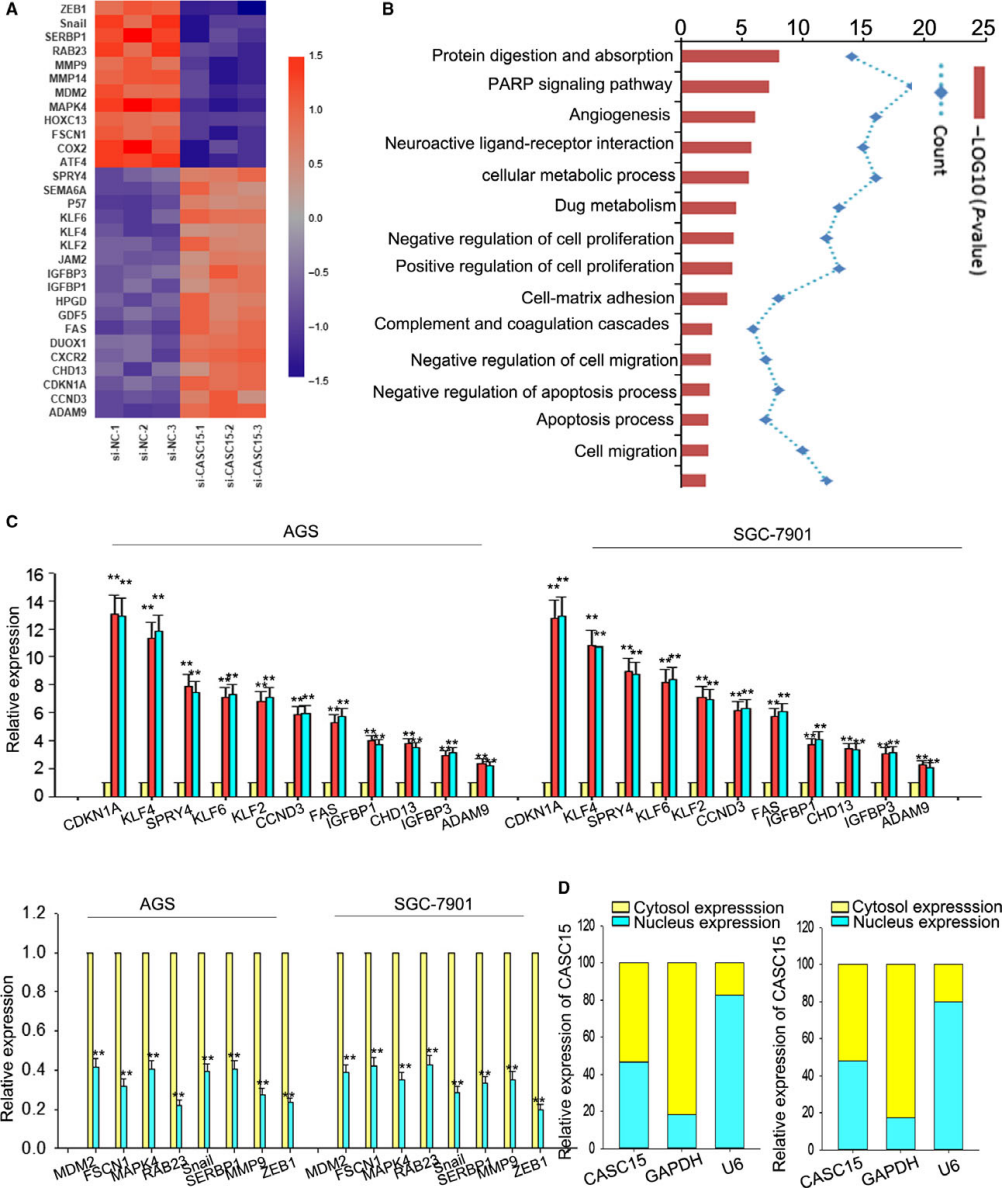
ZEB1 and CDKN1A genes are closely associated with CASC15. (A) Gene expression profiles of AGS cell in response to the knockdown of CASC15 by applying RNA transcriptome sequencing. (B) Gene ontology analysis revealed that these genes were involved in many signal pathways, including cell proliferation and cell migration. (C) qRT‐PCR showed that all these genes could be significantly affected by CASC15; with ZEB1 and CDKN1A exhibiting the greatest change. (D) Subcellular fractionation location assays indicated that CASC15 was located in both the nucleus and cytoplasm in AGS and SGC7901 cells. Error bars represent the mean ± SD of at least three independent experiments. **P <* *0.01 vs. control group.

To probe the possible pathway involved in CASC15, we first evaluated the gene expression changes of AGS cell after the knockdown of CASC15 by applying RNA transcriptome sequencing. A total of 793 mRNA were up‐regulated (FC ≥ 2.0) and 479 mRNA down‐regulated (Fig. 4A). The gene ontology technique was used to analyze these genes. As demonstrated in Fig. 4B, these genes were involved in many signal pathways including cell proliferation and cell migration. Among these genes related to migration and proliferation, we selected those with a relatively high fold change in response to the knockdown of CASC15 (KLF4, KLF2, KLF6, SPRY4, CDKN1A, CCND3, FAS, CHD13, IGFBP1, IGFBP3, ADAM9, MDM2, MMP9, ZEB1, Snail, SERBP1, MAPK4, RAB23, FSCN1) for further study.

To confirm the effect of CASC15, the expressions of these genes were examined in AGS and SGC7901 cells using qRT‐PCR. All these genes could be significantly affected by CASC15 (Fig. 4C), with ZEB1 and CDKN1A exhibiting the greatest change. To probe the potentially regulated mechanism, the expression of CASC15 in cytoplasm vs. nucleus was detected. By applying subcellular fractionation location assays, we found that the level of CASC15 was equally distributed in nucleus and cytoplasm in AGS and SGC7901 cells (Fig. 4D), implying that CASC15 exerted its effect at both transcriptional and post‐transcriptional levels.

Currently, there is accumulating evidence from studies that many functions of lncRNA are realized through interaction with chromatin‐modifying enzymes to activate epigenetic functions or silence gene expressions (Marchese and Huarte, 2014). For instance, PRC2, consisting of EZH2, SUZ12 and EED, is a methyl‐transferase which can epigenetically regulate gene expression through catalyzing the di‐ and trimethylation of lysine residue 27 of histone 3 (H3K27me3) (Cao *et al*., 2002; Yuan *et al*., 2012). The interaction between lncRNA and PRC2 has also been revealed in many types of cancers, such as colorectal cancer (Ding *et al*., 2017), non‐small‐cell lung cancer (Sun *et al*., 2016a) and GC (Sun *et al*., 2016b).

**Figure 5:**
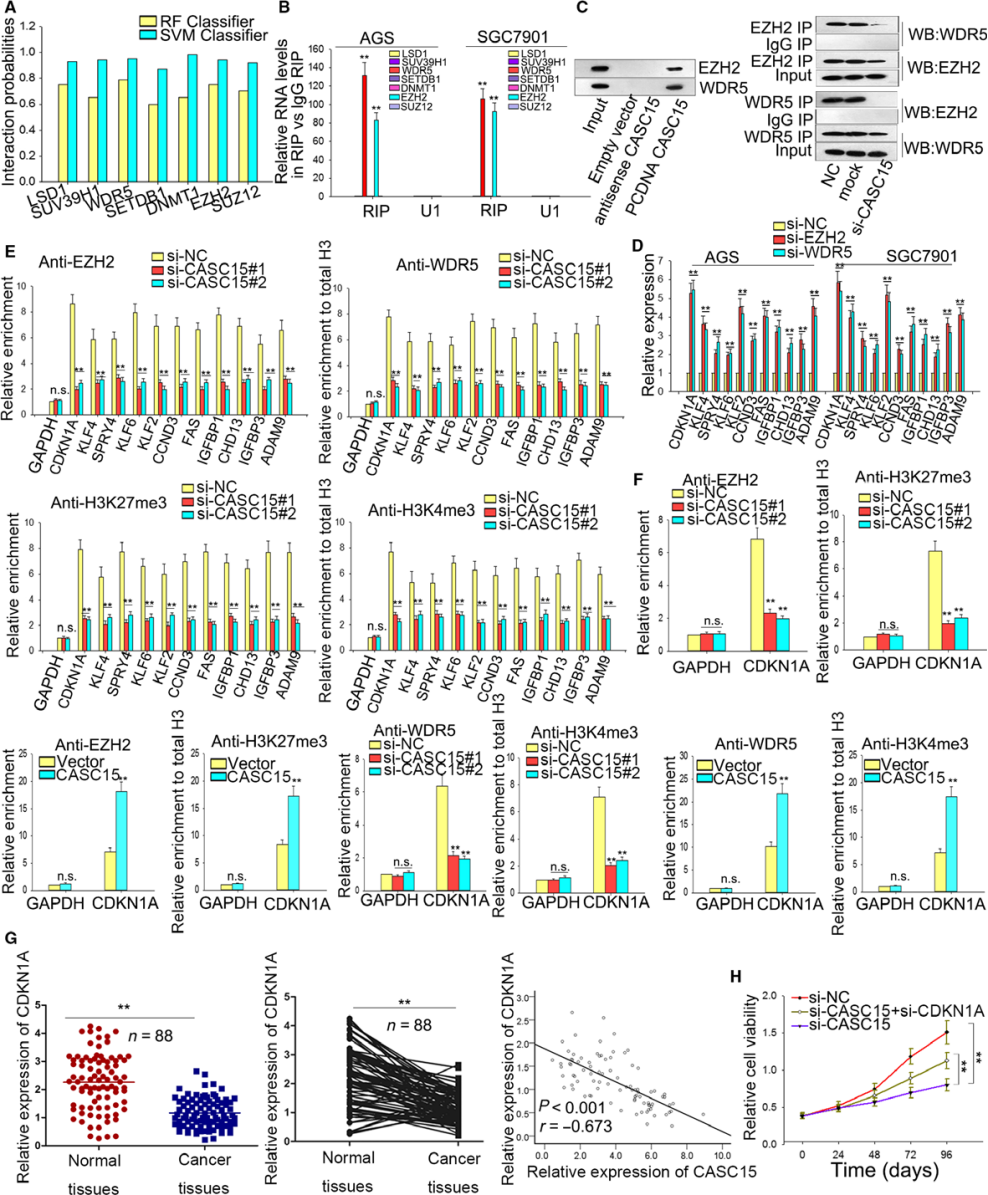
CASC15 modulates CDKN1A through interaction with EZH2 and WDR5 in nucleus. (A) The online bioinformatics analysis was employed to predict the potential chromatin modifiers. (B) RIP was applied to confirm the interaction of chromatin modifiers with the antibodies of these predicted chromatin modifiers. (C) RNA pull‐down assays showed that the labeled CASC15 could pull down EZH2 and WDR5 from the nuclear extract fraction of AGS cell. IP ssay illustrated that CASC15 was required to bridge the interaction between EZH2 and WDR5. (D) qRT‐PCR demonstrated that the silencing of EZH2 or WDR5 increased the expression levels of suppressed genes. (E) ChIP assays revealed that knockdown of CASC15 weakened the binding of EZH2/WDR5 and H3K27me3/H3K4me3 levels across the promoters of these CASC15‐mediated suppression genes. (F) ChIP indicated that CDKN1A was a *bona fide* target of CASC15‐regulated genes. (G) qRT‐PCR and Spearman's correlation analysis revealed that CDKN1A was decreased in GC tissues and was negatively correlated with CASC15. (H) Rescue assays revealed that down‐regulated CASC15‐mediated growth inhibition could be reversed by silenced CDKN1A. Error bars represent the mean ± SD of at least three independent experiments. **P <* *0.01 vs. control group.

To determine the mechanism for CASC15‐mediated gene modulation, we employed the online bioinformatics analysis (http://pridb.gdcb.iastate.edu/RPISeq/) to predict the potential chromatin modifiers. LSD1 (H3K4me3), SETDB1 (H3K9me3), WDR5 (H3K4me3), SUV39H1 (H3K9me3), DNMT1, EZH2 (H3K27me3) and SUZ12 (H3K27me3) positively interacted with chromatin modifiers (Fig. 5A). RIP was used to confirm the interaction of chromatin modifiers with the antibodies of these predicted chromatin modifiers. EZH2 and WDR5 were involved in the enrichment in RIP, with U1 as the negative control (Fig. 5B). The WD repeat domain 5 (WDR5), a key subunit of MLL1, has been proven to interact with numerous lncRNA, indicating the existence of an oncogenic role in tumorigenesis through mediation of H3K4 trimethylation (Chen *et al*., 2015; Yang *et al*., 2014). Thus, it was reasonable to hypothesize that CASC15 might serve as a scaffold to recruit EZH2 and WDR5. To prove this hypothesis, we performed RNA pull‐down assays showing that none of the labeled CASC15, either the empty vector or the anti‐sense CASC15, was able to pull‐down EZH2 or WDR5 from the nuclear extract fraction of AGS cell (Fig. 5C). Results from IP showed that EZH2 IP could retrieve WDR5 and, in turn, WSR5 IP could retrieve EZH2 from AGS cells, whereas knockdown of CASC15 weakened the cooperation of EZH2 with WDR5, hinting that CASC15 was necessary to facilitate the interaction between EZH2 and WDR5 (Fig. 5C).

We then silenced EZH2 and WDR5 to determine the roles of EZH2/WDR5 in co‐regulating CASC15‐mediated gene suppression. We found that after silencing of EZH2 or WDR5, the expression levels of suppressed genes were also increased (Fig. 5D). Additionally, ChIP assays revealed that silenced CASC15 weakened the H3K27me3/H3K4me3 levels of EZH2/WDR5 and decreased the binding of EZH2/WDR5 to the promoter of genes which could suppressed by CASC15 (Fig. 5E). Among these genes, CDKN1A presented the highest fold change. The lncRNA‐mediated hypermethylation in the promoter region of CDKN1A has been proven to be capable of causing the transcriptional inactivation of CDKN1A, which was demonstrated in GC progression (Liu *et al*., 2017a,b). Results from ChIP showed that silencing of CASC15 significantly reduced the sponging of EZH2/WDR5 and H3K27 trimethylation/H3K4 trimethylation levels by the promoter region of CDKN1A, whereas overexpressed CASC15 increased the sponging of EZH2/WDR5 and H3K27 trimethylation/H3K4 trimethylation levels, indicating that CDKN1A was a *bona fide* target of genes modulated by CASC15 (Fig. 5F). To confirm this, the level of CDKN1A and its relation to CASC15 in tissues was explored using qRT‐PCR. CDKN1A was down‐regulated in GC tissues in comparison with the matched healthy tissues and was negatively related to the level of CASC15 (Fig. 5G). The rescue assays revealed that down‐regulated CASC15‐mediated growth inhibition could be reversed by silenced CDKN1A (Fig. 5H). These results suggested that CASC15 modulated GC cell growth at least partially by epigenetically silencing CDKN1A by acting as a molecular sponge for EZH2 and WDR5 in nucleus.

### 3.5 CASC15 modulates the level of ZEB1 through sponging miRNA‐33a‐5p in cytoplasm

**Figure 6:**
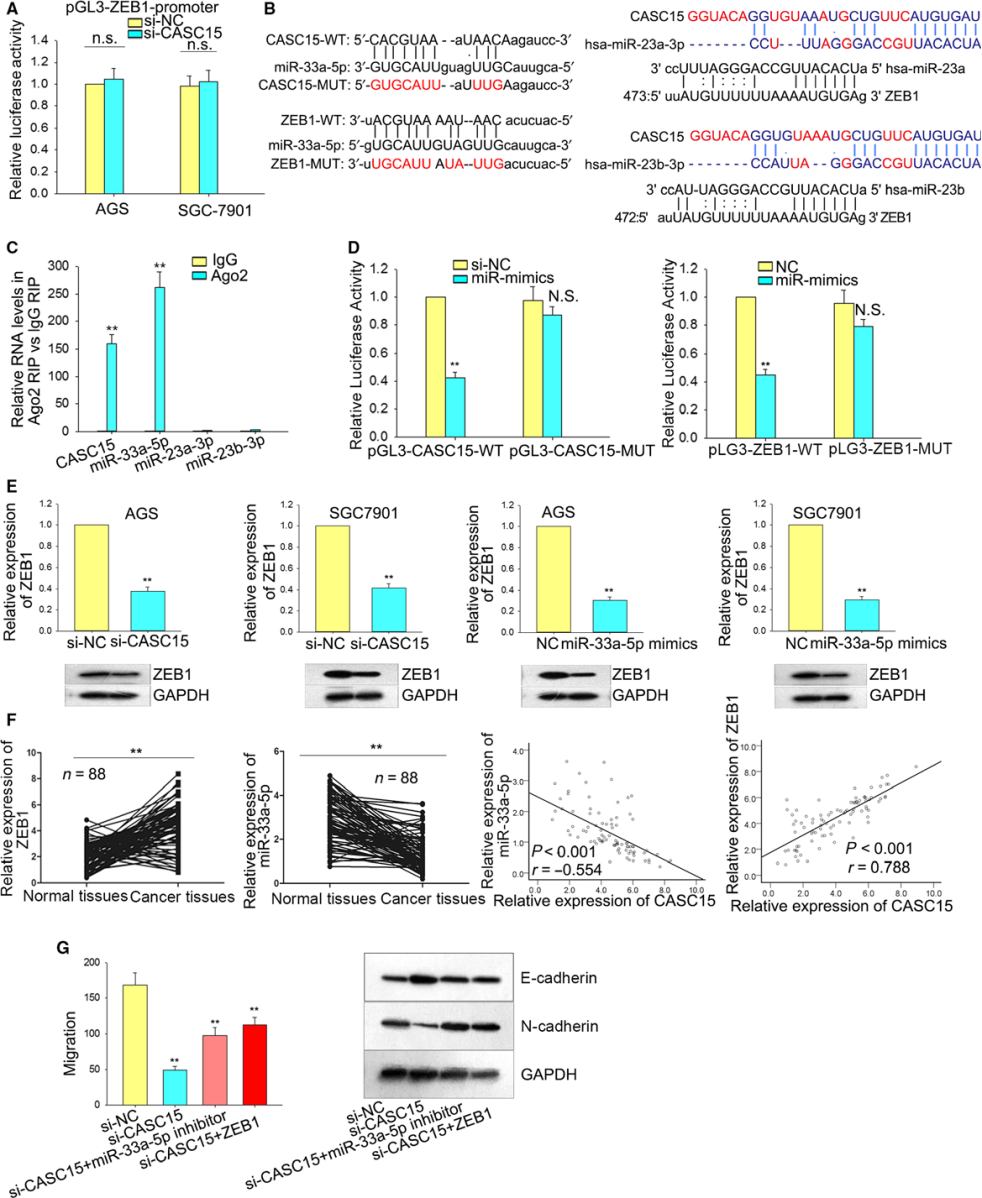
CASC15 modulates the expression of ZEB1 through sponging miRNA‐33a‐5p in cytoplasm. (A) Luciferase reporter assays demonstrated that CASC15 could not impact the transactivation of ZEB1 promoter, suggesting that CASC15 might regulate ZEB1 mRNA at a post‐transcriptional level. (B) Binding sites between CASC15 and miR‐33a‐5p, and between ZEB1 and miR‐33a‐5p. (C) RIP assays revealed that miR‐33a‐5p was involved in the CASC15‐mediated ZEB1 modulation. (D) Luciferase reporter assays demonstrated that both CASC15 and miR‐33a‐5p were able to influence the luciferase activity of ZEB1. (E) qRT‐PCR and western blot analysis showed that silenced CASC15 or forced expression of miR‐33a‐5p decreased ZEB1 expression levels in AGS and SGC7901 cells. (F) qRT‐PCR and Spearman's correlation analysis showed that miR‐33a‐5p/ZEB1 were respectively significantly down‐regulated/up‐regulated in the GC tissues and negatively/positively correlated with CASC15. (G) Rescue experiments revealed that silenced CASC15‐mediated migration inhibition and EMT process blockage could be reversed by inhibiting miR‐33a‐5p or forcibly expressing ZEB1. Error bars represent the mean ± SD of at least three independent experiments. **P <* *0.01 vs. control group.

Results from RNA‐Seq revealed that ZEB1 could be significantly decreased when CASC15 was silenced. ZEB1, a critical regulator in EMT formation, was a member of the most common genes with expression changes in solid tumors. To determine the regulatory mechanism of CASC15 on ZEB1, we first conducted a luciferase reporter assay to observe how CASC15 affected the promoter of ZEB1. CASC15 could not impact the transactivation of ZEB1 promoter, suggesting that CASC15 might regulate ZEB1 mRNA at post‐transcriptional level (Fig. 6A). Recently, it has been demonstrated that lncRNA can function as a molecular sponge to compete with miRNA for the shared mRNA‐responding element (MRE), which is harbored by the miRNA (Cesana *et al*., 2011). To examine the possible interaction of miRNA, we applied online bioinformatics analysis (http://carolina.imis.athena-innovation.gr/diana_tools/web/index.php?r=site%2Findex and http://www.microrna.org/microrna/home.do) to predict the interaction. Based on the results from online bioinformatics analysis, we selected three miRNA (hsa‐miR‐23a‐3p, hsa‐miR‐23b‐3p and miR‐33a‐5p) which were possibly bonded with both CASC15 and ZEB1 (Fig. 6B). For further verification, we performed RIP assays with Ago2 antibodies. In addition to CASC15, miR‐33a‐5p, not miR‐23a‐3p or miR‐23b‐3p, was enriched in Ago2‐containing beads, indicating that miR‐33a‐5p was involved in the CASC15‐mediated ZEB1 modulation (Fig. 6C). To confirm this, luciferase reporter analyses were carried out. Co‐transfection of AGS and SGC‐7901 cells with pGL3‐CASC15‐WT vector and miR‐33a‐5p mimicks the significantly impaired luciferase reporter activity of WT‐CASC15, as compared with cells transfected with pGL3‐CASC15‐MUT and miR‐NC (Fig. 6D). We then constructed luciferase reporter vectors covering the 3′‐UTR of ZEB1; this revealed that miR‐33a‐5p mimicks the significantly inhibited luciferase reporter activity of ZEB1. Conversely, mutation of the nucleotides in miR‐33a‐5p putative targeting sites resulted in compete abrogation of the repressive effect (Fig. 6D). To prove that CASC15 could really act as a ceRNA, we additionally performed RNA pull‐down assays with biotin‐labeled miR‐33a‐5p, miR‐23a‐3p and miR‐23b‐3p oligos. It was observed that of the three miRNA, only biotin‐labeled miR‐33a‐5p oligos pulled down CASC15, rather than the mutated oligos, demonstrating that CASC15 acted as a ceRNA to bind to miR‐33a‐5p (Fig. S2A). Quantitative RT‐PCR and western blot analysis showed that silenced CASC15 or forced expression of miR‐33a‐5p decreased ZEB1 expression levels in AGS and SGC7901 cells (Fig. 6E). Additionally, results from qRT‐PCR revealed that the expressions of miR‐33a‐5p/ZEB1 were respectively significantly decreased/increased in the GC tissues and negatively/positively correlated with the expression level of CASC15 (Fig. 6F).

To further confirm the relation among CASC15, miR‐33a‐5p and ZEB1, we designed rescue assays, which indicated that the decreased expression of ZEB1 mediated by silenced CASC15 was slightly recovered by suppressed miR‐33a‐5p (Fig. S2B). Such a rescue assay demonstrated the positive correlation between CASC15 and ZEB1, and the negative correlation between miR‐33a‐5p and ZEB1. Finally, the rescue experiments revealed that silenced CASC15‐mediated migration inhibition and EMT process blockage could be reversed by inhibiting miR‐33a‐5p or forcibly expressing ZEB1 (Fig. 6G).

Due to the fact that miR‐33a‐5p was down‐regulated in GC tissues and was able rescue si‐CASC15‐mediated suppression of EMT formation, the effect of miR‐33a‐5p on the EMT process was examined. It was evident that the up‐regulation of miR‐33a‐5p improved the protein level of E‐cadherin but decreased that of N‐cadherin, in comparison with the control group (Fig. S2C). These results suggested that CASC15 modulated GC cell migration at least in part by acting as a ceRNA, competitively binding with ZEB1 for the MRE of miR‐33a‐5p in cytoplasm.

## 4 Discussion

Gastric cancer is one of the predominant causes of cancer death in China (Chen *et al*., 2016; Torre *et al*., 2015). Despite great improvements in therapies for advanced and malignant cancers, metastasis and invasion remain large threats for cancer fatalities and are among the most uncontrollable aspects of GC (Chaffer and Weinberg, 2011; Hanahan and Weinberg, 2011; The Cancer Genome Atlas Research Network, 2014). It would therefore be a great help to determine the molecules for cancer treatment and to remove the obstacles in the way of clinical therapy in order to figure out the interactions among these pathways (Steeg, 2006).

It has been shown that lncRNA is involved in cellular programming, including cell apoptosis, proliferation, migration and invasion (Guttman *et al*., 2011; Wang and Chang, 2011; Wang *et al*., 2014). It has also been revealed that lncRNA plays a role as key regulators for GC migration (Lai *et al*., 2014; Wiestler *et al*., 2014; Yang *et al*., 2015). The lncRNA can exert diverse manipulatory roles, including in chromatin modification, RNA programming, localization and translation, and can even act as competitive endogenous RNA (Cesana *et al*., 2011; Heo and Sung, 2011; Xing *et al*., 2014). Although there have been multiple papers introducing different functions of lncRNA in cancers, information about lncRNA CASC15 in GC is not yet clear.

The aim of this study was to analyze in detail the biological significance of CASC15 for GC diagnosis and later‐stage therapy. Specifically, CASC15 was highly expressed in GC tissues and cells, predicting a poor prognosis for GC patients. In addition, functional assays were applied to test the effects of silenced CASC15 and strengthened CASC15 on the biological behaviors in GC, demonstrating that the over‐ or underexpression of CASC15 remarkably suppressed or boosted cell proliferation through inducing cell cycle arrest and apoptosis. In addition, western blot showed that the expression of the epithelial marker (E‐cadherin) and the mesenchymal marker (N‐cadherin) was regulated by the up‐regulated CASC15 or down‐regulated CASC15, demonstrating that CASC15 could affect cell migratory and invasive abilities by influencing EMT progression. All these findings from the *in vitro* experiments confirmed the oncogenic role of CASC15 in the carcinogenesis of GC. The *in vivo* experiments also demonstrated that the knockdown of CASC15 could weaken tumor volume and weight in nude mice, and influence the EMT process, as confirmed by western blot and IHC assays. Subsequently, mechanistic assays proved that CASC15 is engaged in the tumorigenesis of GC through interaction with EZH2 and WDR5 to modulate CDKN1A in nucleus. It was discovered that the knockdown of CASC15 triggered the silence of ZEB1 in cytoplasm, which was attributed to the competitive sponging of CASC15 with miR‐33a‐5p.

Based on the results from our study, we put forward the following key points to explain the potential mechanism of CASC15 in the progression of GC. The segment of CASC15 in the nucleus suppressed the expression of CDKN1A in an enhancer‐like manner through recruiting EZH2 and WDR5 to the promoter region of CDKN1A, whereas the fraction of CASC15 in the cytoplasm silenced ZEB1 by being a ceRNA to competitively bind to miR‐33a‐5p.

## Author contributions

QW, SX and JM conceived and designed the project; QW and PH acquired the data; QW, TW, and WM analyzed and interpreted the data; QW, MS and YW wrote the paper.

## Supporting information


**Fig. S1.** The effect of silenced CASC15 on cell cycle and apoptosis. (A, left) The knockdown of CASC15 induced cell cycle arrest, increasing cell numbers in G0/G1 phase and decreasing cell numbers in S phase. (A, right) Overexpression of CASC15 accelerated cell cycle progression, both of which were based on flow cytometry analysis of cell cycle. (B, left) Silenced CASC15 increased apoptosis rate. (B, right) Up‐regulated CASC15 impaired cell apoptosis. (B) Both results were based on flow cytometry analysis of apoptosis. (C) On the basis of western blot, (left) silenced CASC15 enhanced the levels of caspase 3 and caspase 9; (right) up‐regulated CASC15 impaired the levels of caspase 3 and caspase 9. Error bars represent the mean ± SD of at least three independent experiments. **P < 0.01 vs. control group.

**Fig. S2.** The relationship among CASC15, miR‐33a‐5p and ZEB1, and the effect of miR‐33a‐5p on EMT formation. (A) RNA pull‐down assays were performed to demonstrate the binding between CASC15 and miR‐33a‐5p. (B) Rescue assays were designed to detect the effect of silenced CASC15 and miR‐33a‐5p on the expression of ZEB1. (C) Western blot was applied to measure the effect of overexpressed miR‐33a‐5p on EMT formation. Error bars represent the mean ± SD of at least three independent experiments. *P < 0.05 and **P < 0.01 vs. control group.
