# Supplementary figures and images for: Long non‐coding RNA CASC15 regulates gastric cancer cell proliferation, migration and epithelial mesenchymal transition by targeting CDKN1A and ZEB1
Source: Mol Oncol. 2018 May 9;12(6):799–813. doi: 10.1002/1878-0261.12187 (PMC5983148; doi:10.1002/1878-0261.12187)

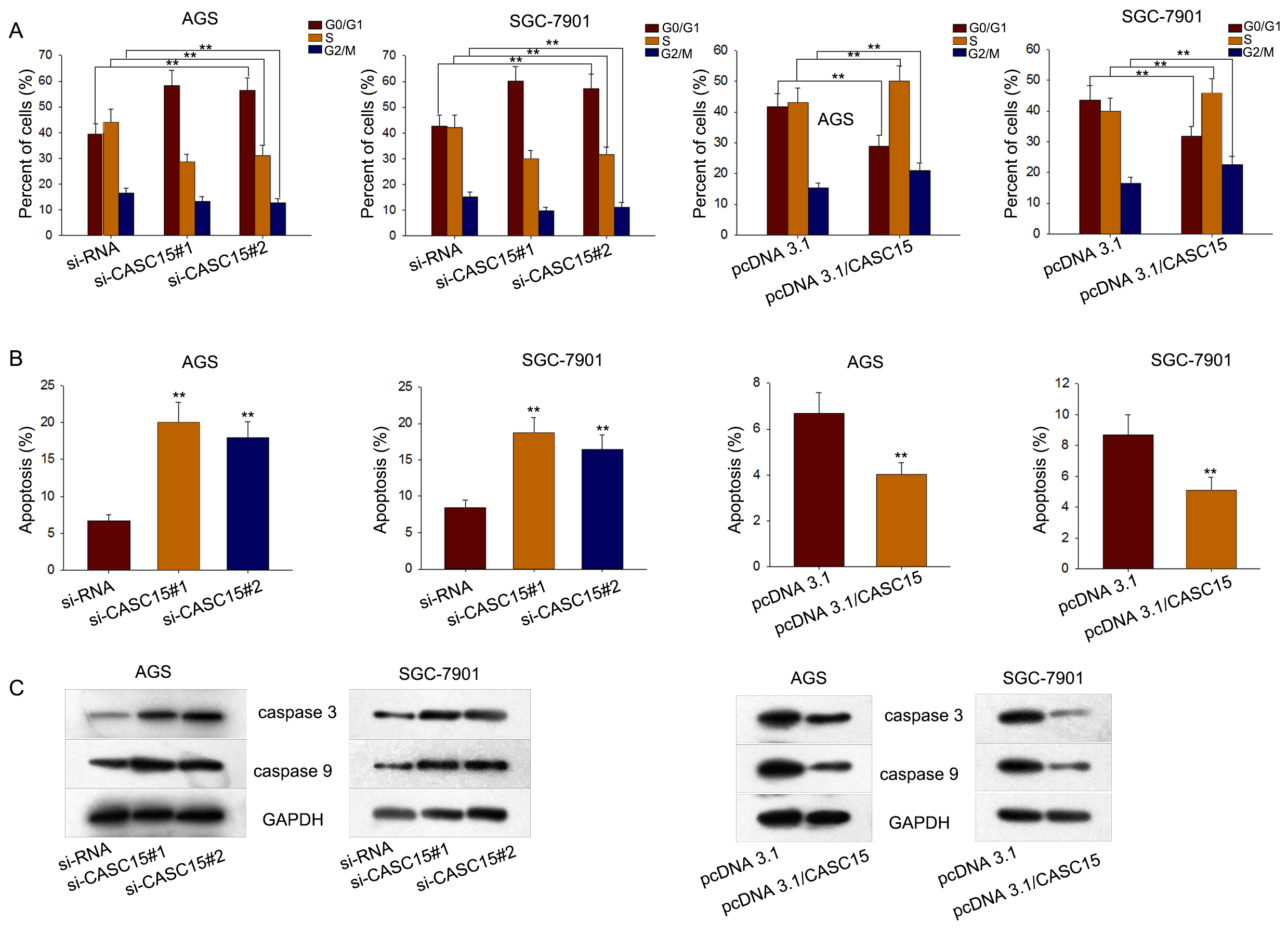

Supplement: Supplementary file 1 — Fig. S1. The effect of silenced CASC15 on cell cycle and apoptosis. (A, left) The knockdown of CASC15 induced cell cycle arrest, increasing cell numbers in G0/G1 phase and decreasing cell numbers in S phase. (A, right) Overexpression of CASC15 accelerated cell cycle progression, both of which were based on flow cytometry analysis of cell cycle. (B, left) Silenced CASC15 increased apoptosis rate. (B, right) Up‐regulated CASC15 impaired cell apoptosis. (B) Both results were based on flow cytometry analysis of apoptosis. (C) On the basis of western blot, (left) silenced CASC15 enhanced the levels of caspase 3 and caspase 9; (right) up‐regulated CASC15 impaired the levels of caspase 3 and caspase 9. Error bars represent the mean ± SD of at least three independent experiments. **P < 0.01 vs. control group. [file MOL2-12-799-s001.tif]

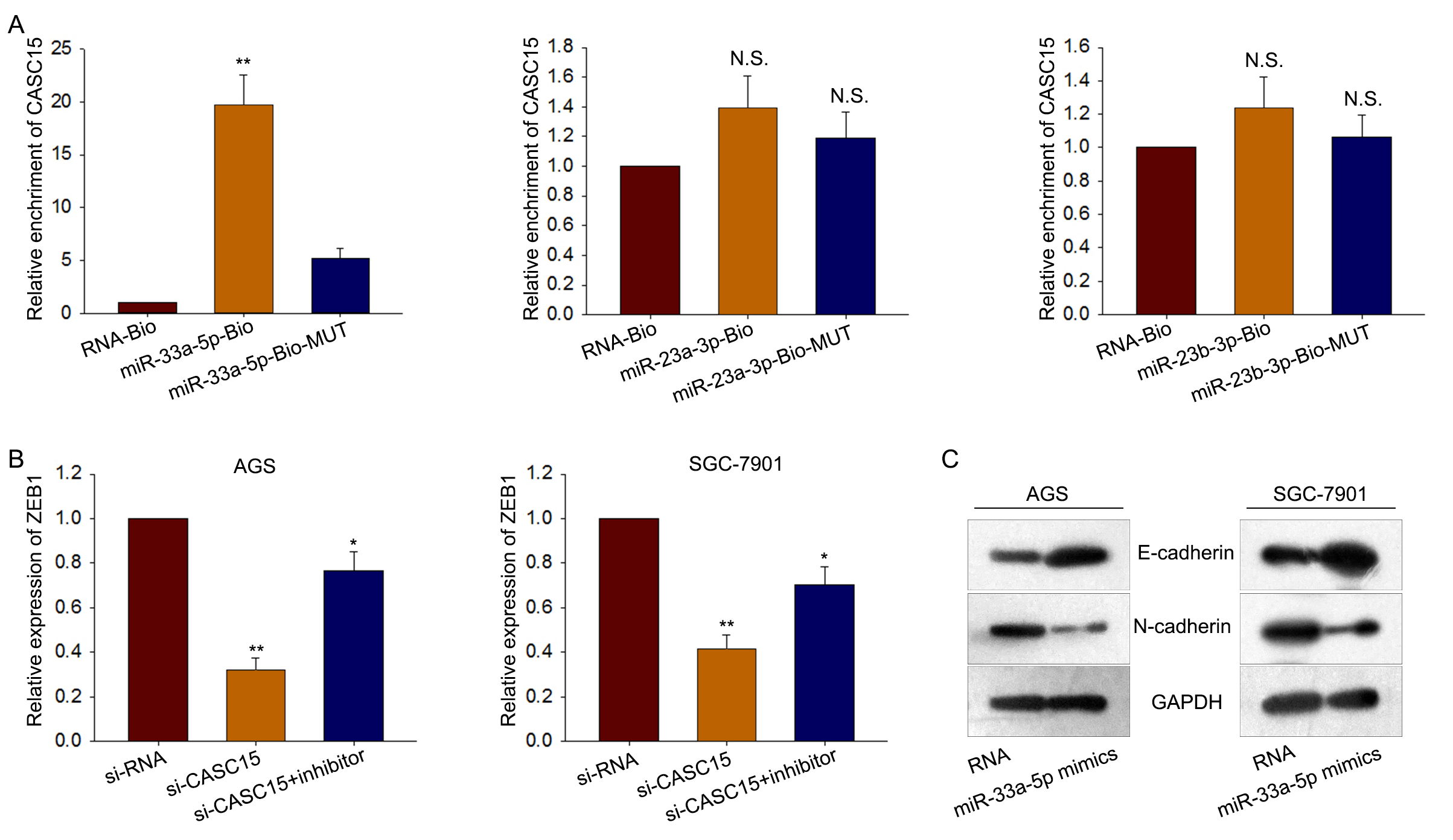

Supplement: Supplementary file 2 — Fig. S2. The relationship among CASC15, miR‐33a‐5p and ZEB1, and the effect of miR‐33a‐5p on EMT formation. (A) RNA pull‐down assays were performed to demonstrate the binding between CASC15 and miR‐33a‐5p. (B) Rescue assays were designed to detect the effect of silenced CASC15 and miR‐33a‐5p on the expression of ZEB1. (C) Western blot was applied to measure the effect of overexpressed miR‐33a‐5p on EMT formation. Error bars represent the mean ± SD of at least three independent experiments. *P < 0.05 and **P < 0.01 vs. control group. [file MOL2-12-799-s002.tif]
